# Biological Activity of Carbazole Alkaloids and Essential Oil of *Murraya koenigii* Against Antibiotic Resistant Microbes and Cancer Cell Lines

**DOI:** 10.3390/molecules16119651

**Published:** 2011-11-21

**Authors:** Thilahgavani Nagappan, Perumal Ramasamy, Mohd Effendy Abdul Wahid, Thirukanthan Chandra Segaran, Charles S. Vairappan

**Affiliations:** 1 Laboratory of Natural Products Chemistry, Institute for Tropical Biology and Conservation, Universiti Malaysia Sabah, 88999 Kota Kinabalu, Sabah, Malaysia; 2 School of Medicine, Universiti Malaysia Sabah, 88999 Kota Kinabalu, Sabah, Malaysia; 3 Institute of Marine Biotechnology, University Malaysia Terengganu, 21030 Kuala Terengganu, Terengganu, Malaysia

**Keywords:** *Murraya koenigii*, carbazole alkaloids, antibiotic resistant, pathogenic bacteria, cancer cell lines

## Abstract

A total of three carbazole alkaloids and essential oil from the leaves of *Murraya koenigii* (Rutaceae) were obtained and examined for their effects on the growth of five antibiotic resistant pathogenic bacteria and three tumor cell lines (MCF-7, P 388 and Hela). The structures of these carbazoles were elucidated based on spectroscopy data and compared with literature data, hence, were identified as mahanine (**1**), mahanimbicine (**2**) and mahanimbine (**3**). The chemical constituents of the essential oil were identified using Gas Chromatography-Mass Spectroscopy (GCMS). These compounds exhibited potent inhibition against antibiotic resistant bacteria such as *Staphylococcus aureus* (210P JTU), *Psedomonas aeruginosa * (ATCC 25619), *Klebsiella pneumonia* (SR1-TU), *Escherchia coli* (NI23 JTU) and *Streptococcus pneumoniae* (SR16677-PRSP) with significant minimum inhibition concentration (MIC) values (25.0–175.0 mg/mL) and minimum bacteriacidal concentrations (MBC) (100.0–500.0 μg/mL). The isolated compounds showed significant antitumor activity against MCF-7, Hela and P388 cell lines. Mahanimbine (**3**) and essential oil in particular showed potent antibacteria and cytotoxic effect with dose dependent trends (≤5.0 μg/mL). The findings from this investigation are the first report of carbazole alkaloids’ potential against antibiotic resistant clinical bacteria, MCF-7 and P388 cell lines.

## 1. Introduction

Two major medical issues in the 21st century are the sudden rise in antibiotic resistant bacteria and the high incidence of cancer [1,2,3]. Emergence of antibiotic-resistant bacteria is often regarded as an inevitable phenomenon due to the immediate availability of good healthcare system and antibiotics. The surge of antibiotic resistant microbes cannot be taken lightly, as they have started to show resistance against vancomycin, which was regarded as the last resort in clinical practice [4]. This has become a strong catalyst in reigniting a frantic search for novel metabolites with potent antimicrobial potential [5].

In the last few decades, reports on carcinoma cases have shown a drastic increase, although there have been significant advancements in cancer treatment. Changes in lifestyle and environment are regarded as the leading causes, resulting in over 10 million reported cases globally in year 2001 [6]. Two of the most common type of cancer in females are cervical and breast cancer, with the mortality rate for cervical cancer in the United States in 2006 being 37%. This surge in mortality is also attributed to resistance against treatment with cisplatin [5]. Breast cancer on the other hand, has become the most commonly diagnosed malignant tumor in women, accounting for 24% of all female cancer and being the second most lethal cancer among women [7,8]. On the other hand, leukemia is a common hemato-oncological disorder that affects different age groups with the chronic phase of leukemia affecting mostly adults [9]. The common correlation between these three malignancies is that they tend to relapse and develop drug resistance towards cancer treatments. As such, there is a strong need for the establishment of new chemopreventives and the discovery of new drugs to combat these carcinomas [6,10].

The quest for new antimicrobial and chemopreventive drugs based on traditional herbs is of great interest [1]. The carbazole alkaloids of *M. koenigii* from the family Rutaceae have been reported to show potent cytotoxic activity against human leukemia cells, prostate cancer cell lines, viral and clinical pathogens [11,12,13,14,15]. However, no information is available pertaining to their activity against clinical antibiotic resistant bacteria and breast cancer cell lines (MCF-7). As stated above, these two medical problems are on the rise and there is an urgent need to find sources of alternative lead drugs in an effort to supplement the ones available. Therefore, we embarked on an investigation of the possible usage of *M. koengii* to address these issues. This report presents the pioneering findings on the potent bioactive activity of *M. koenigii*’s chemicals, particularly against clinical antibiotic resistant bacteria strains and the MCF7, P388 cell lines.

## 2. Results and Discussion

### 2.1. Structure Elucidation of Alkaloids from the Leaves of Murraya koenigii

A dark green ethanol extract was obtained from 270 g of leaves and an aliquot (5 g) was subjected to silica gel flush column chromatography. Six fractions were obtained by gradient elution using a hexane-ethyl acetate solvent system. Fractions were spotted on silica gel normal phase Thin Layer Chromatography plates (Merck, Germany), and visualized under UV light at 254 nm and using molybdo-phosphoric acid spray. Fractions with secondary metabolites were subjected to semi-preparative HPLC to isolate the alkaloids. A total of three major alkaloids were isolated from *M. koenigii*, namely compound **1** (0.40%) from fraction 3, while compounds **2** (0.24%) and **3** (0.66%) were isolated from fraction 2. The isolated compounds were subjected to spectroscopic measurements and the resulting data are given in Table 1.

**Table 1:** ^1^H-NMR and ^13^C-NMR spectral data of the isolated compounds from leaves of *Murraya koenigii* (recorded at 600/150 MHz in CD_3_OD; δ in ppm).

Position	Compound 1		Compound 2		Compound 3	
	δ_C_	δ_H_	δ_C_	δ_H_	δ_C_	δ_H_
1	117.5	6.60	118.8	6.57	118.3	6.84
2	129.2	5.64	126.7	5.67	128.9	5.63
3	79.3	-	79.6	-	79.3	-
4	149.9	-	152.9	-	150.9	-
5	118.3	-	109.7	6.84	119.3	-
6	120.6	7.48	120.1	7.71	121.8	7.61
6a	116.7	-	118.9	-	117.9	-
7	119.6	7.64	119.3	7.69	119.7	7.86
7a	118.1	-	125.3	-	124.8	-
8	109.1	6.62	129.3	-	119.9	7.06
9	156.5	-	125.6	7.09	125.5	7.23
10	97.9	6.81	111.5	7.27	111.4	7.36
10a	143.3	-	140.2	-	141.6	-
11a	136.9	-	138.7	-	136.9	-
11b	105.8	-	106.0	-	105.5	-
3-CH_3_	26.0	1.41	26.6	1.41	26.4	1.41
5-CH_3_	16.4	2.27	-	-	16.2	2.29
8-CH_3_	-	-	21.7	2.46	-	-
9-OH	-	5.01	-	-	-	-
1′-CH_2_	42.2	1.72–1.78	42.3	1.70–1.74	42.1	1.71–1.73
2′-CH_2_	24.1	2.17–2.20	24.0	2.14–2.18	23.9	2.15–2.19
3′	125.7	5.12	122.4	5.12	124.9	5.10
4′	132.2	-	132.6	-	132.3	-
4′-CH_3_	17.8	1.64	17.8	1.64	17.6	1.63
4′-CH_3_	26.0	1.56	26.0	1.57	25.8	1.55
NH	-	7.65	-	7.78	-	7.85

**Figure 1:**
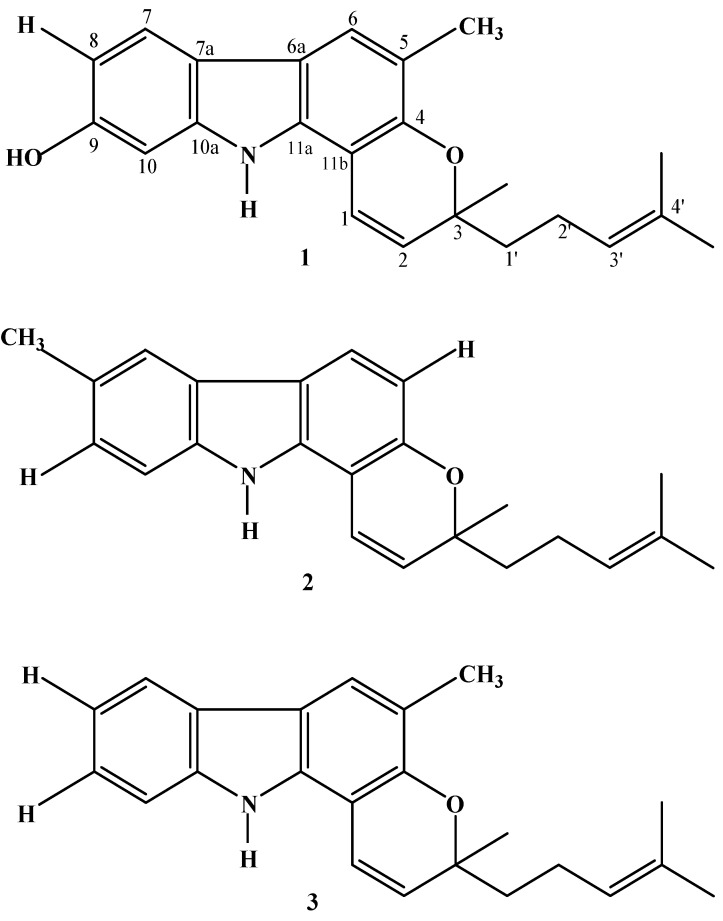
Chemical structures of carbazole alkaloids [mahanine (**1**), mahanimbicine (**2**), mahanimbine (**3**)] isolated from leaves of *Murraya koenigii *(L) Spreng.

Based on independent structure elucidation the structures of these three compounds were determined as mahanine (**1**) with the molecular formula, C_23_H_25_NO_2_, electron impact mass spectra (EIMS) (70 eV *m/z*) mass value of 347 [M]^+^ (100), 332 (19), 304 (8), 278 (15), 264 (75) and optical rotation, [α]_D_^25^ = + 7.6° (CHCl_3_; *c* 5.38); mahanimbicine (**2**) with the molecular formula, C_23_H_25_NO, electron impact mass spectra (EIMS)(70 eV *m/z*) mass value of 331[M]^+^ (23), 316 (6), 248 (100), 210 (3) and optical rotation, [α]_D_^25^ = + 58.1° (CHCl_3_; *c* 0.26); mahanimbine (**3**) with the molecular formula C_23_H_25_NO, electron impact mass spectra (EIMS)(70 eV *m/z*) mass value of 331[M]^+^ (24), 248 (100), 210 (5) and optical rotation, [α]_D_^25^ = + 30.0° (CHCl_3_; *c* 0.69). Further, data comparison with published data confirmed them to be known carbazole alkaloids, whose structures are shown in Figure 1.

### 2.2. Profiling of Essential Oil from Leaves of Murraya koenigii

The volatile aromatic hydrocarbons were identified based on their Retention Indices (AART) and mass fragment pattern with reference to the NIST 08 and FFNSC version 1.2 databases. A total of 34 aromatic volatile constituents were identified from the oil of *M. koenigii*, where two sesquiterpene hydrocarbons, β-caryophyllene (19.50%) and α-humulene (15.24%) were present as the major volatile metabolites. Detailed analysis revealed that the 34 volatile constituents could be further grouped into 12 oxygenated monoterpenes, 12 sesquiterpene hydrocarbons, nine oxygenated sesquiterpenes and one oxygenated diterpene. The identified volatile constituents from oils of *M. koenigii* investigated are presented in Table 2.

**Table 2:** Composition (%) of volatile compounds in essential oil of *Murraya koenigii *from Sabah, Malaysia.

RT (min)	Ref RI	RI	Volatile compound	Concentration (%)
15.73	1082 ^a^	1079	Linalol	0.56
15.91	1099 ^b^	1095	*trans*-Sabinene hydrate	0.53
17.01	1109 ^a^	1112	*trans*-2-Cyclohexen-1-ol	0.48
17.88	1110 ^a^	1113	*cis*-2-Cyclohexen-1-ol	0.54
19.70	1189 ^b^	1185	*para*-Cymen-8-ol	10.31
20.42	1143 ^b^	1139	β-Terpineol	2.52
21.03	1175 ^a^	1170	*trans*-Piperitol	0.40
21.74	1276 ^a^	1273	Chrysanthenyl acetate	0.39
24.16	1284 ^b^	1279	Lavandulyl acetate	1.67
24.37	1285 ^b^	1285	Bornyl acetate	1.68
28.31	1375 ^b^	1370	α-Copaene	0.82
28.91	1390 ^b^	1385	β-Elemene	0.35
29.39	1394 ^a^	1390	( *Z*)-Jasmone	0.11
30.29	1494 ^a^	1489	β-Caryophyllene	19.50
31.09	1438 ^b^	1436	Aromadendrene	0.72
31.84	1454 ^b^	1448	α-Humulene	15.24
32.70	1420 ^a^	1425	Butanedioic acid	2.18
33.29	1487 ^b^	1480	β-Selinene	3.81
33.30	1470 ^a^	1472	Naphthalene	1.90
33.55	1474 ^a^	1478	α-Selinene	6.10
34.37	1518 ^b^	1512	δ-Cadinene	2.03
36.03	1562 ^b^	1566	Nerolidol	2.64
36.05	1564 ^b^	1569	*trans*-Nerolidol	1.32
36.28	1475 ^a^	1481	Cycloheptane	0.13
36.92	1576 ^b^	1580	Spathulenol	1.98
37.13	1587 ^b^	1591	Caryophyllene oxide	2.14
37.26	1594 ^b^	1590	Viridiflorol	1.51
38.13	1598 ^a^	1592	2-Naphthalenemethanol	0.66
38.26	1079 ^b^	1074	Trivertal	0.35
38.55	1696 ^b^	1694	Juniper camphor	1.57
38.83	1581 ^b^	1579	Cubenol	0.57
39.44	1472 ^a^	1476	β-Cadina-1(6),4-diene	0.50
40.16	1593 ^a^	1596	Selina-6-en-4-ol	4.78
54.95	2106 ^b^	2105	Phytol	10.07
**Composition of grouped volatile compounds (%)**				
*Monoterpenes (oxygenated)*				35.29
*Sesquiterpenes (hydrocarbon)*				35.29
*Sesquiterpenes (oxygenated)*				26.47
*Diterpenes (oxygenated)*				2.94

* Identification of volatile components is based on mass spectra value in reference to NIST 08 (^a^) and FFNSC Ver.1.2 (^b^) standard libraries.

### 2.3. Antibacterial Activity

Results on antibacterial activities involving the evaluation of inhibition zones, minimum inhibition concentrations (MICs) and minimum bacteriacidal concentrations (MBCs) of the isolated carbazole alkaloids and essential oil are shown in Table 3.

**Table 3:** Antibacterial properties of mahanine (**1**), mahanimbicine (**2**), mahanimbine (**3**) and essential oil tested on five strains of clinical human pathogens.

Antibacterial Properties	Compounds	Tested Bacteria				
		Sa	Pa	Kp	Ec	Sp
**DIZ (mm) (mean ± SD)**	**1**	18.5 ± 0.5	18.5 ± 0.5	14.5 ± 1.0	12.5 ± 0.5	18.0 ± 1.0
	**2**	16.0 ± 0.5	12.5 ± 0.5	18.5 ± 0.5	14.0 ± 0.5	11.0 ± 0.5
	**3**	8.5 ± 0.5	NT	10.5 ± 0.5	10.5 ± 0.5	8.0 ± 0.5
	**EO**	12.5 ± 0.5	16.5 ± 0.5	18.5 ± 1.0	14.5 ± 0.5	10.0 ± 1.0
**MIC (mg/mL)**	**1**	25.0	25.0	50.0	75.0	12.5
	**2**	25.0	50.0	50.0	25.0	25.0
	**3**	75.0	NT	125.0	150.0	175.0
	**EO**	50.0	25.0	25.0	50.0	75.0
**MBC (** **μg/mL)**	**1**	300.0	300.0	325.0	250.0	100.0
	**2**	>500	325.0	250.0	200.0	150.0
	**3**	>500	NT	325.0	>500	250.0
	**EO**	>500	250.0	>500	325.0	200.0

Note: Sa-*Staphylococcus aureus* (210P JTU); Pa-*Pseudomonas aeruginosa* (ATCC25619); Kp-*Klebsiella pneumoniae* (SR1-TU); Ec-*Escherichia coli *(NI23 JTU); Sp-*Streptococcus pneumoniae* (SR16677-PRSP). The DIZ value of negative control for each bacterium was 4.6 mm (bored well diameter in the agar plates). The concentration of DIZ test were 30 mg/mL. P1: mahanine, P2: mahanimbicine, P3: mahanimbine, EO: essential oil.

Selective antibacterial activities were exhibited by the carbazole alkaloids although they have similar chemical skeletons. Overall, the diameter of inhibition zone for all the tested compounds fell in the range of 8.0 mm to 18.0 mm. When examined against *S. aureus*, *P. aeruginosa* and *S. pneumoniae*, mahanine (**1**) exhibits the highest susceptibility with inhibition zone of 18.5 ± 0.5 mm, 18.5 ± 0.5 mm and 18.0 ± 0.5 mm respectively while mahanimbicine (**2**) specifically exhibits the highest susceptibility against *K. pneumonia*, with an inhibition zone of 18.5 ± 0.5 mm. Mahanimbine (**3**) appear to be less susceptible to antibiotic resistance bacteria strains as its inhibition zones were in the range of 8.0 mm to 10.0 mm. The minimum inhibition concentration (MIC) of these carbazole alkaloids ranges between 12.5 mg/mL to 175.0 mg/mL. A minimum of 12.5 mg/mL of mahanine (**1**) was observed as the MIC against *S. pneumoniae* whereas mahanimbicine (**2**) exhibits a moderate MIC of 25.0 mg/mL against *S. aureus*, *E. coli* and *S. pneumoniae*, respectively. Meanwhile, the minimum bacteriacidal concentration (MBC) (μg/mL) values of these carbazole alkaloids ranged between 125.0 μg/mL to 500.0 μg/mL. The MBCs of mahanine (**1**), mahanimbicine (**2**) and mahanimbine (**3**) against *S. pneumoniae* were 100.0 μg/mL, 150.0 μg/mL, 250.0 μg/mL, respectively. Based on this finding, it can be concluded that mahanine (**1**) was most effective in combating antibiotic resistant clinical bacteria followed by mahanimbicine (**2**) and mahanimbine (**3**).

As the essential oil of *M. koenigii* is a mixture of volatile bioactive terpene classes, positive inhibition were observed according to bacterial strains studied. Diameter of inhibition of *M. koenigii* essential oil range between 10.00 mm to 18.50 mm as it was most effective in inhibiting *S. pneumoniae*. The MIC of the essential oil was moderate compared to the MIC values of the individual carbazole alkaloids. The minimal value of inhibition (MIC) was 25.00 μg/mL against *P. aeruginosa *and *K. pneumoniae* while 200.00 μg/mL was the minimal bactericidal concentration (MBC) against *S. pneumoniae*.

### 2.4. Anti-Tumor Properties of Isolated Carbazole Alkaloids and Essential Oil

**Figure 2:**
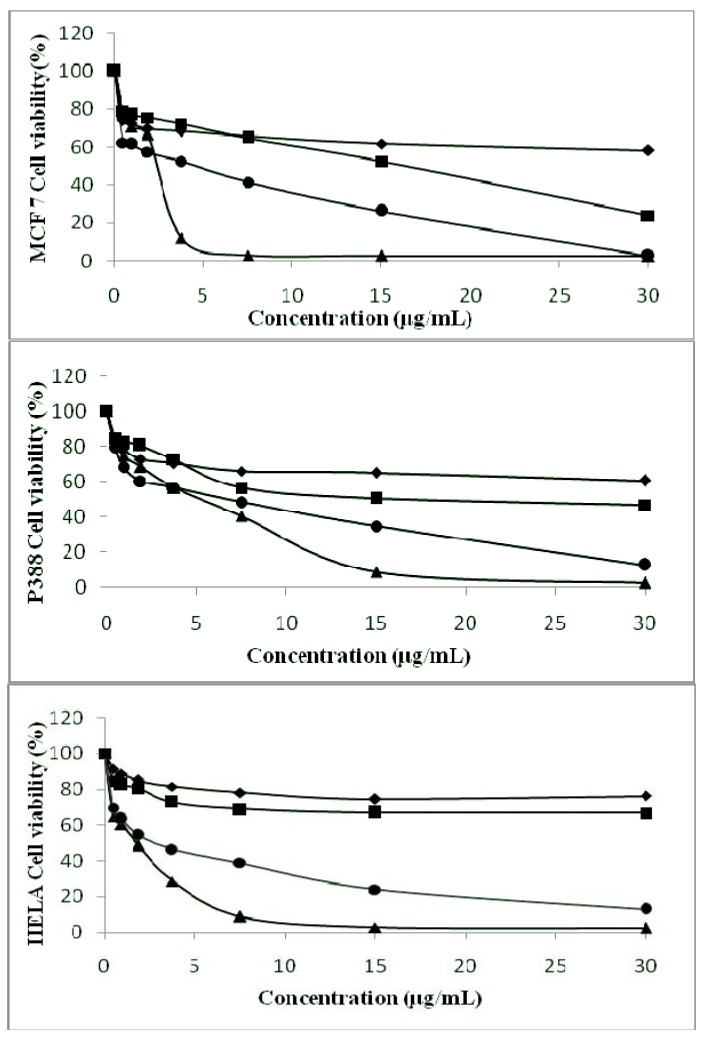
Effect of mahanine (**1**) (♦), mahanimbicine (**2**) ( ■), essential oil (•) and mahanimbine (**3**) (▲) on the growth of MCF-7 cells, P388 cells and HELA cells according to concentration tested.

The MTT assay is a simple and reliable technique to measure cell viability used for screening of anti-proliferative agents. Figure 2 presents the summarized plots of cell viability (%) *versus* concentrations of carbazole alkaloids and essential oil analyzed.

The results showed that all the carbazole alkaloids and essential oil exhibit anti-proliferative effects against the three cell lines in dose-dependent manner although taking into account that the growth rate of cancer cells differs. It was found that higher the concentration of compound tested, the lower the cell viability percentages were. Overall, mahanimbine (**3**) and essential oil of *M. koenigii* were found to inhibit the proliferation of the cell lines. Mahanimbine (**3**) was found to exhibit significant cytotoxic activity against all the cancer cell lines studied, with half inhibition concentration (IC_50_) values of 2.12 μg/mL, 5.00 μg/mL, 1.98 μg/mL in the MCF-7, P388 and HeLa cell lines, respectively. Meanwhile, essential oil of *M. koenigii* displays IC_50_ values of 6.00 μg/mL, 7.01 μg/mL, 2.83 μg/mL in the MCF-7, P388 and HeLa cell lines, respectively. Mahanimbicine (**2**) exhibits a moderate level of cytotoxicity with the IC_50_ values of 17.00 μg/mL and 18.31 μg/mL against the MCF-7 cell line and P388 cell line. In comparison, mahanine (**1**) were found to be less cytotoxic against all the cell line studied as the IC_50_ values fall beyond 30.00 μg/mL.

Carbazole alkaloids from the genus *Murraya* are also well known for their phytomedicine properties as astringents, analgesic, antidysentric, treatment for snake bites, influenza, rheumatism and traumatic injury [12,19,20]. In addition, this investigation has shown that carbazole alkaloids and essential oil from Malaysian *M. koenigii *have antibacterial potential against antibiotic resistant bacteria strains. Despite having the similar chemical skeletons, these carbazole alkaloids displayed selective antibacterial and antitumor activity due to their different side chain moieties. Results of antibacterial activity revealed mahanine (**1**) is the most effective in inhibiting antibiotic resistant bacteria while mahanimbine (**3**) was found to be the most cytotoxic against the tested cancer cell lines. Positioning of functional groups (-H, -OH and -CH_3_) in the chemical skeleton could be responsible for these selective bioactive attributes [21]. Generally, the bioactivity of carbazoles were reported to decrease with the introduction of an oxygen atom on the carbazole nucleus, however this effect can be reversed by the replacement of the hydrogen atom of the hydroxyl group by an alkyl or alkoxy group, especially acetyl and methyl rest [22,23,24]. This might explain why the positioning of -CH_3_ in the mahanimbine (**3**) skeleton displayed a vital role in its selective antitumor activity [25,26]. To the best of our knowledge, this is the first report on the bioactive potential of mahanimbine (**3**) against the MCF-7 cell line. This result could be used as a basis for the development of a structure-orientated drug discovery program. In addition, it has provided an important indication that mahanimbine (**3**) should be further explored as a possible chemotherapeutic agent in human breast cancer studies. 

Essential oils of plants in the family Rutaceae are often composed of mono- and sesquiterpenes. Monoterpenes and sesquiterpenes derivatives such as linalool, terpineol, camphor, selinene are reported to possess antibacterial, insect-repelling and toxic activities [27]. As such, the presence of high concentrations of β-caryophyllene (19.50%) along with caryophyllene oxide (2.16%) in the oil of *M. koenigii *clearly contributes to its bacteria susceptibility properties [28,29] as the activity exhibited could also be explained as a synergistic effect of the dominant volatile constituents [30]. Previous investigations [28,29,30,31] have explained that the antimicrobial activity of the essential oils derived from plants are found to be more pronounced against Gram-positive than against Gram-negative bacteria. This action is often correlated to the presence of terpene constituents although the mechanism of action of compounds from this class against Gram-negative bacteria is not fully understood, but the absence of an outer phospholipidic membrane in Gram-positive bacteria allows the penetration of essential oil’s bioactive hydrocarbons that can cause leakage of vital intracellular constituents and impairment of the bacterial enzyme systems [32].

Classes of terpenes are also known for their antitumor attributes. Studies report that the volatile sesquiterpene hydrocarbons α-humulene, β-caryophyllene and α-caryophyllene isolated from the family Rutaceae are active against human alveolar basal epithelial cells (A-549), colon carcinoma cells (DLD-1) and human prostate adenocarcinoma (LNCaP) cell lines as well as possessing anti-proliferative ability towards myeloid leukemia (K562) cells. Ironically, constituents of monoterpene groups, such as β-pinene, γ-terpinene and ρ-cymene were found to be inactive against DLD-1 cancer cell lines [28]. This could explain the synergism of active hydrocarbon sesquiterpenes and oxygenated sesquiterpenes, which constitute 61.76% of the volatile chemical found in oil of *M. koenigii*, responsible for the anti-proliferation of HeLa, P388 and MCF-7 cell lines. Reports have suggested [29] that oils that have IC_50_ values less than 0.13 mg/mL could be possible candidates for further development as cancer therapeutic agent. As such, essential oil of *M. koenigii* could be such a possible candidate given its IC_50_ value ranging between 2.83 μg/mL to 7.01 μg/mL.

## 3. Experimental

### 3.1. Plant Material

Pest free leaves of *Murraya koenigii* (L.) Spreng (Rutaceae) were collected from Kg. Bobot, Kota Belud, Sabah in August 2010. Voucher specimens were deposited at the BORNEENSIS, Herbarium of the Institute for Tropical Biology and Conservation, Universiti Malaysia Sabah (BORH 37581).

### 3.2. Extraction and Identification

Air-dried *M. koenigii *leaves (270 g) were extracted with ethanol (3 liter) (Merck, Germany) for 7 days using a soxhlet apparatus. Extract were concentrated under reduced pressure at 40 °C to yield a dark green extract (14.75 g). A portion (5 g) was then subjected to successive silica gel (Merck, Kiesegel 60, 70–230 mesh) column chromatography with a stepwise gradient of hexane-ethyl acetate (hex-EtOAc; 9:1, 8:2, 7:3, 6:4, 1:1) before the column was washed with chloroform-methanol-water (65:25:4) to obtain six fractions of different polarity. Fractions were then profiled using a high performance liquid chromatography (HPLC) apparatus equipped with a UV-Vis detector monitored at 254 nm (Prominence, Shimadzu, Japan) coupled with a reverse phase Phenomenex C-18 ODS (10 mm × 250 mm × 5 μm) column separation. Mobile phase in gradient elution was: A: 50% MeCN: 50% H_2_O, B: 100% MeCN with the profile of: 0 min–30 min: A: 30% B: 70%, 30.01 min–60.00 min A: 0% B: 100% and flow rate was set at 2.00 mL/min under 40 °C. A manual injection of 50 μL of pre-filtered solution (0.2 μm nylon membrane syringe filters, Whatman) was analyzed.

### 3.3. Essential Oil Extraction and Analysis

Selected pest-free leaves of *M. koenigii* (50 g) were chopped and subjected to hydro-distillation using a Clevenger-type apparatus for a duration of 8 h. Yield of oil based on fresh weight was 0.12%. Distilled oil was collected in pentane, dried over anhydrous Na_2_SO_4_, concentrated *in vacuo*, stored in air-tight glass vials flushed with nitrogen (N_2_) gas and kept at −20 °C prior to analysis. Analysis of the essential oil was performed using a Shimadzu QP-2010 gas chromatograph coupled with a Shimadzu GCMSQP-2010Plus detector (Shimadzu, Japan) using a SGE BPX-5 (30.0 m × 0.25 μm i.d., film thickness 0.25 μm) fused silica capillary column. High purity helium was used as the carrier gas at a constant flow rate of 0.8 mL/min. A total of 1 μL sample was injected (split ratio 100:1) into GCMS using AOC5000 auto injector for analysis. The initial temperature was set at 50 °C, heated at a rate of 3 °C/min to 280 °C and held isothermally for 5 min. Ion source temperature for these analysis was set at 200 °C, while the interface temperature was set at 280 °C and the mass spectrometer was set to operate in electron ionization mode with an ionizing energy of 70 eV as acquisition mass range from 40 a.m.u to 450 a.m.u. at 0.25 scan/s. Identification of volatile organic constituents was confirmed using published electron impact-mass spectra (EI-MS) in the National Institute for Standard and Technology (NIST) 1998 and Shimadzu’s Flavours and Fragrance of Natural and Synthetic Compounds (FFNSC) version 1.2 computerized mass spectral libraries. The retention indices were determined based on a homologous series of *n*-alkanes (C_8_–C_40_; Custom Retention Time Index Standard, Restek Corp, USA) external standard analyzed under the same operating conditions and calibrated based on the Automatic Adjustment of Compound Retention Time (AART) function of the GCMS. Relative concentrations of the essential oil components were calculated based on GC peak area with the AART correction factors.

### 3.4. Isolation and Structure Elucidation of Alkaloids

Carbazole alkaloids were isolated based on their Thin Layer Chromatography (TLC) and High Performance Liquid Chromatography profile data. Presence of alkaloids was detected on TLC upon spraying with Dragendoff’s reagent and isolation of the respective fractions was done using semi-preparative HPLC using a C-18 ODS column (Inertsil ODS-3, 20 mm × 250 mm × 5 μm) coupled with a UV-Vis detector (Prominence, Shimadzu Corp., Japan) monitored at 254 nm. Gradient elution conditions were; A: 50% MeCN: 50% dH_2_O and B: 100% MeCN under the profile of; 0 min–30 min: A: 30% B: 70%, 30.01 min–60.00 min A: 0% B: 100% with flow rate of 6.00 mL/min with injection volume of 200 μL at 40 °C. All solutions were pre-filtered using Whatman 13 mm, 0.2 μm nylon membrane syringe filters before injections. A total of three major peaks were isolated from Fraction 2 and Fraction 3.

Isolated peaks were subjected to TLC confirmation with Drangendoff spray and ^1^H-NMR revealed the purity of the isolate and confirmed the identity of the alkaloids with the presence of aromatic and amine protons. Three compounds were isolated and subjected to ^1^H-, ^13^C- and 2D NMR spectroscopic analyses. The structures of compounds **1**–**3** were determined based on the comparison of their ^1^H- and ^13^C-NMR data with those reported in the literature [28,29,30,31]. All spectral data were obtained on the following instruments; IR on a ThermoNicolet FT-IR spectrometer, optical rotations was measured on an AUTOPOL IV automatic polarimeter (Rudolph Research Analytical), ^1^H-NMR (600 MHz) and ^13^C-NMR (150 MHz) were recorded with a JEOL ECA 600 (Japan Electronic Optics Laboratory Co. Ltd., Tokyo, Japan) spectrometer, with TMS as internal standard. HR-ESI-TOFMS data were obtained using LCMS-IT-TOF (Shimadzu).

### 3.5. Cell Culture and Assay

#### 3.5.1. Antibacterial Activity Assay

Antibacterial activity was assayed with the standard agar well diffusion method (NCCLS, 2000). The bacterial suspension (10^6^ CFU/mL) was “flood-inoculated” onto the surface of PCA medium and wells (4.6 mm) were cut from the agar. Test compounds and standards were dissolved in DMSO, sterilized by filtration through 0.22 mm sterilizing Milipore express filter (Millex-GP, Bedford, OH, USA) and 60 μL of these solutions were delivered into the wells. Gentamicin (600 mg/well) was used as positive reference standards to determine the sensitivity of each microbial species tested and DMSO solutions were applied as negative controls. The inoculated plates were incubated at 37 °C for 24–48 h. The diameter of inhibition zone (DIZ) of the tested bacteria was measured and expressed in millimeters to evaluate the antibacterial activity of the samples. Tests were performed in triplicates.

#### 3.5.2. Minimum Inhibitory Concentration (MIC) Assay

MIC was assayed using two-fold microdilution broth method (NCCLS, 2003). Dilutions were used to dispense 0.1 mL into each of the sterile 96 wells of a standard tray. Each well contained 5 × 10^5^ colony forming units (CFU/mL) of test bacteria, serially diluted samples and respective growth medium. Negative and positive controls were prepared accordingly using DMSO and gentamicin, respectively. After incubated at 37 °C for 24 h, the microdilution trays were checked with unaided eyes to detect the growth inhibition of the bacteria. The growth end points were determined by comparing the amount of growth in the wells containing test samples with that in the control wells. The acceptable growth (≥2 mm button or definite turbidity) must occur in the positive control well. When a single skipped well occurred, the highest MIC was read. Tests were performed in triplicate for each test concentration.

#### 3.5.3. Minimum Bactericidal Concentration (MBC) Assay

A method in ASM Pocket Guide to Clinical Microbiology was slightly modified to determine the MBC values. Briefly, samples (50 μL) were taken from the wells of the MIC assays, where no visible turbidity (growth) was observed, and spread on freshly prepared PCA plates. The plates were incubated at 37 °C for 24 h so as to determine the MBC, which was defined as the lowest concentration of the samples which allowed less than 0.1% of the original inoculums treated with the compounds to survive and grow on the surface of the medium used. Tests were performed in triplicate.

### 3.6. Anti-Tumor Assay

#### 3.6.1. Cell Lines and Cultivation Conditions

The three cell lines used in this investigation comprised human breast (MCF-7), human cervical (HeLa) and murine leukemia cell lines (P388). The MCF-7, HeLa and P388 cells each were cultured as monolayers in RPMI-1640, supplemented with 10.0% (v/v) heat-inactivated FBS, 100 U/mL penicillin and 100.0 μg/mL streptomycin. All cell cultures were grown in a humidified incubator at 37 °C in 5.0% CO_2_ and 95% O_2_.

#### 3.6.2. Measurement of Cell Viability by MTT Assay

The cytotoxic effects of mahanine (**1**), mahanimbicine (**2**), mahanimbine (**3**) and essential oil were determined by measuring conversion of 3-(4,5-dimethylthiazol-2-yl)-2,5-diphenyltetrazolium bromide (MTT) dye. All pure compounds and essential oil were dissolved in DMSO to a final concentration of 30 μg/mL. Briefly, 2.0 × 10^4^ MCF-7 cells were treated in triplicate with each pure compound and the essential oil. The same treatments were applied on 5.0 × 10^5^ HeLa cells and 1.0 × 10^5^ P388 cells. MTT reagent (5.0 mg/mL) was added and cells were incubated in the dark at 37 °C. DMSO was added to dissolve purple formazon crystals and a microtiter plate reader (Tecan, Switzerland) was used to measure the absorbance at 570 nm with 630 nm as the reference wavelength.

### 3.7. Statistical Analysis

The significance of differences was estimated using Student’s t-test. A p-value of less than 0.05 was considered significant.

## 4. Conclusions

In conclusion, this study has revealed the bioactive potentials of carbazole alkaloids from *M. koenigii *where mahanine (**1**) and mahanimbicine (**2**) inhibited antibiotic resistant bacteria and mahanimbine (**3**) was found to significantly suppress the proliferation of MCF-7 cells. The differences in biological activities could be correlated to differences in their functionality since the chemical skeletons was similar. To the best of our knowledge, this report is the first pertaining to the selectivity of these metabolites towards antibiotic resistant bacteria and MCF-7 cells. This information could be of importance in the development of new antibacterial and antitumor lead metabolites.
